# The common inflammatory etiology of depression and cognitive impairment: a therapeutic target

**DOI:** 10.1186/s12974-014-0151-1

**Published:** 2014-09-02

**Authors:** David J Allison, David S Ditor

**Affiliations:** Department of Kinesiology, Faculty of Applied Health Science, Brock University, 500 Glenridge Ave, St. Catharines, ON L2S 3A1 Canada

**Keywords:** Indoleamine 2,3-dioxygenase, Tryptophan 2,3-dioxygenase, Kynurenine pathway, Chronic Inflammation, Depression, Cognitive impairment, Exercise

## Abstract

Chronic inflammation has been shown to contribute to the development of a wide variety of disorders by means of a number of proposed mechanisms. Depression and cognitive impairment are two such disorders which may share a closely linked inflammatory etiology. The ability of inflammatory mediators to alter the activity of enzymes, from key metabolic pathways, may help explain the connection between these disorders. The chronic up-regulation of the kynurenine pathway results in an imbalance in critical neuroactive compounds involving the reduction of tryptophan and elevation of tryptophan metabolites. Such imbalances have established implications in both depression and cognitive impairment. This may implicate the immune system as a potential therapeutic target in the treatment of these disorders. The most common treatment modalities currently utilized, involve drug interventions which act on downstream targets. Such treatments help to reestablish protein balances, but fail to treat the inflammatory basis of the disorder. The use of anti-inflammatory interventions, such as regular exercise, may therefore, contribute to the effectiveness of current drug interventions in the treatment of both depression and cognitive impairment.

## Background

Chronic inflammation plays an increasingly appreciated role in the pathogenesis of a number of neurological and behavioral disorders including depression and cognitive impairment [1-3]. In addition, chronic inflammation contributes to the pathogenesis of a number of related metabolic disorders, and these disorders in turn have been shown to contribute to the elevated inflammatory state, creating a vicious cycle [4-8]. Consequently, as conditions such as obesity, cardiovascular disease, and diabetes become ever more prevalent, so too does the occurrence of chronic inflammation. Communicatory pathways between systems allow for immune dysfunction to contribute to both neural and endocrinal impairment via a number of inflammatory mechanisms. Proinflammatory mediators possess the ability to directly influence the nervous system by acting on vagal afferents [9], or by crossing the blood brain barrier (BBB) either through leaky sites at the circumventricular organs [10], or via specialized active transporters [11]. Proinflammatory cytokines have also been shown to influence hormone secretion by acting directly on receptors within the hypothalamic-pituitary-adrenal (HPA) axis [12]. Alternatively, a number of cytokines have been shown to indirectly influence neural and endocrinal disorders by altering the regulation of enzymes. This may result in a shift in key metabolic pathways resulting in an imbalance in critical neuroactive compounds.

Both depression and cognitive impairment may share a closely linked inflammatory etiology stemming from a cytokine-induced imbalance in the kynurenine pathway. As this pathway provides the primary route for tryptophan (TRP) degradation, it plays a major role not only in the maintenance of serotonin (5-HT) synthesis, but also in the critical balance between neurotoxic and neuroprotective metabolites. As such, a state of chronic inflammation, as is commonly reported in cases of depression and severe cognitive deficits, may contribute to the pathogenesis of each of these disorders [13-16].

Consequently, the kynurenine pathway has become a prospective target for treatment interventions. However, the majority of current intervention strategies for depression and cognitive impairment utilize drugs which target downstream enzymes, transporters, and receptors. Although such treatments are valuable, there may also be merit in implementing intervention strategies which target the inflammatory basis of such disorders. Alternative strategies with proven anti-inflammatory benefits, such as regular exercise, may provide a simple and effective long-term strategy in the treatment and prevention of associated disorders by influencing enzyme function and aiding in the reestablishment of critical protein balances.

### The kynurenine pathway

The kynurenine (KYN) pathway involves a cascade of enzymatic steps responsible for the degradation of tryptophan (TRP) into a number of metabolites, known as kynurenines. This pathway is responsible for approximately 95% of whole body TRP metabolism, and is of critical importance concerning the maintenance of several key amino acids with neuromodulatory roles [17,18]. As TRP is the precursor for 5-HT synthesis within the brain, the maintenance of sufficient levels has important implications concerning depression. Additionally, several TRP metabolites such quinolinic acid (QUIN) and kynurenic acid (KYNA) have roles in symptoms of both depression and cognitive impairment, further stressing the importance of a strictly controlled rate of TRP metabolism.

**Figure 1:**
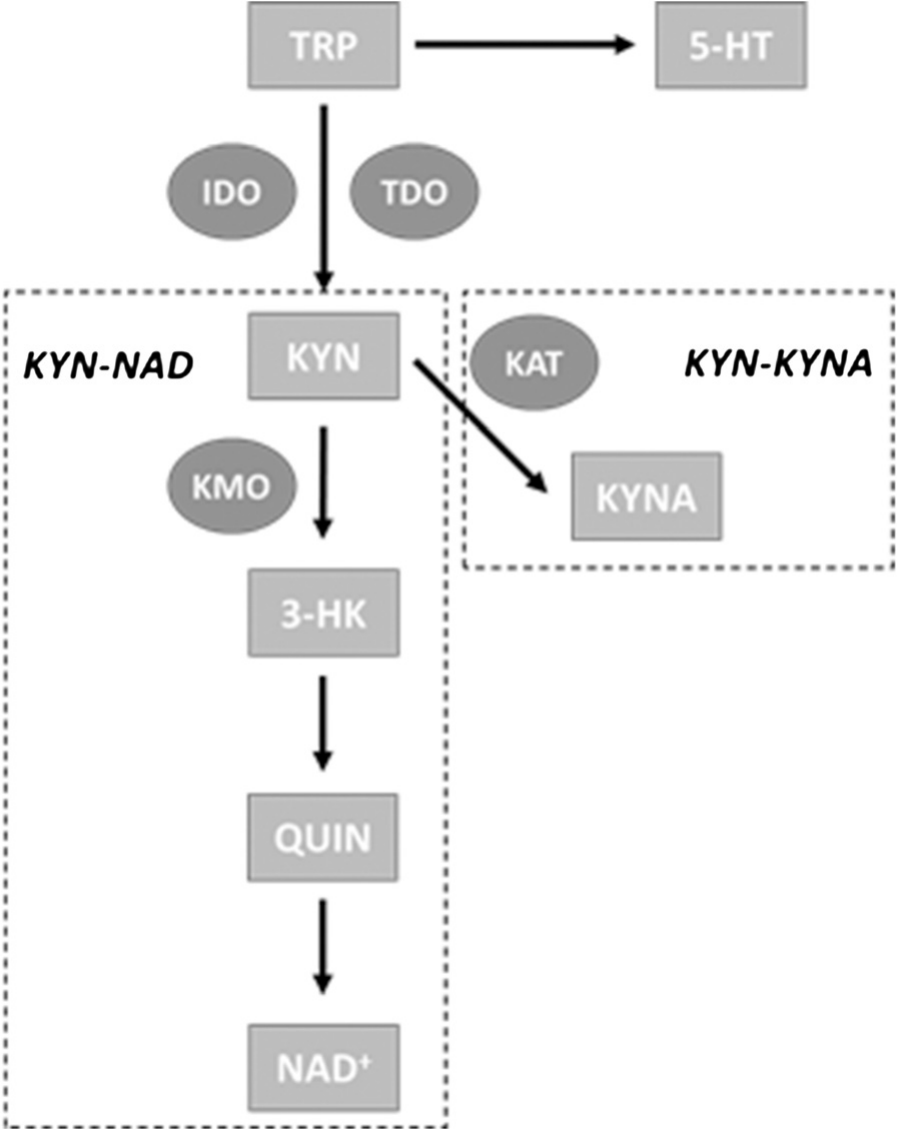
**Simplified depiction of tryptophan breakdown in the kynurenine pathway.** Tryptophan (TRP) that is not transported across the blood brain barrier (BBB) for the synthesis of serotonin (5-HT) is degraded into kynurenine (KYN) by the enzymes indoleamine 2,3-dioxygenase (IDO) and tryptophan 2,3-dioxygenase (TDO). After this point, KYN is further degraded along one of two distinct branches; either the kynurenine-nicotinamide adenine dinucleotide (KYN-NAD) branch, or the kyneine-kynurenic acid (KYN-KYNA) branch. Within the KYN-NAD branch, KYN is acted on by the enzyme kynurenine 3-monooxygenase (KMO) whereby it is converted to 3-hydroxykynurenine (3-HK) and later quinolinic acid (QUIN) via a spontaneous reaction (and ultimately NAD^+^). Within the KYN-KYNA branch, KYN is acted on by the enzyme kynurenine aminotransferase (KAT) whereby it is converted to kynurenic acid (KYNA).

The degradation of TRP is controlled by two rate-limiting enzymes, known as indoleamine 2,3-dioxygenase (IDO), and tryptophan 2,3-dioxygenase (TDO) [19,20]. Together, under the regulation of cytokines, steroids, and growth factors, these enzymes control the conversion of TRP into the first metabolite of the kynurenine pathway; kynurenine (KYN). This metabolite is then further metabolized along one of two distinct branches of this enzymatic cascade including the kynurenine-kynurenic acid (KYN-KYNA) branch and the kynurenine-nicotinamide adenine dinucleotide (KYN-NAD) branch [19]. Within the former branch, KYN is further converted to the metabolite kynurenic acid (KYNA) via the enzyme kynurenine aminotransferase (KAT). The latter branch utilizes the enzyme kynurenine 3-monooxygenase (KMO) to convert KYN to the metabolite 3-hydroxykynurenine (3-HK) and further in the cascade, quinolinic acid (QUIN) (see Figure 1).

**Figure 2:**
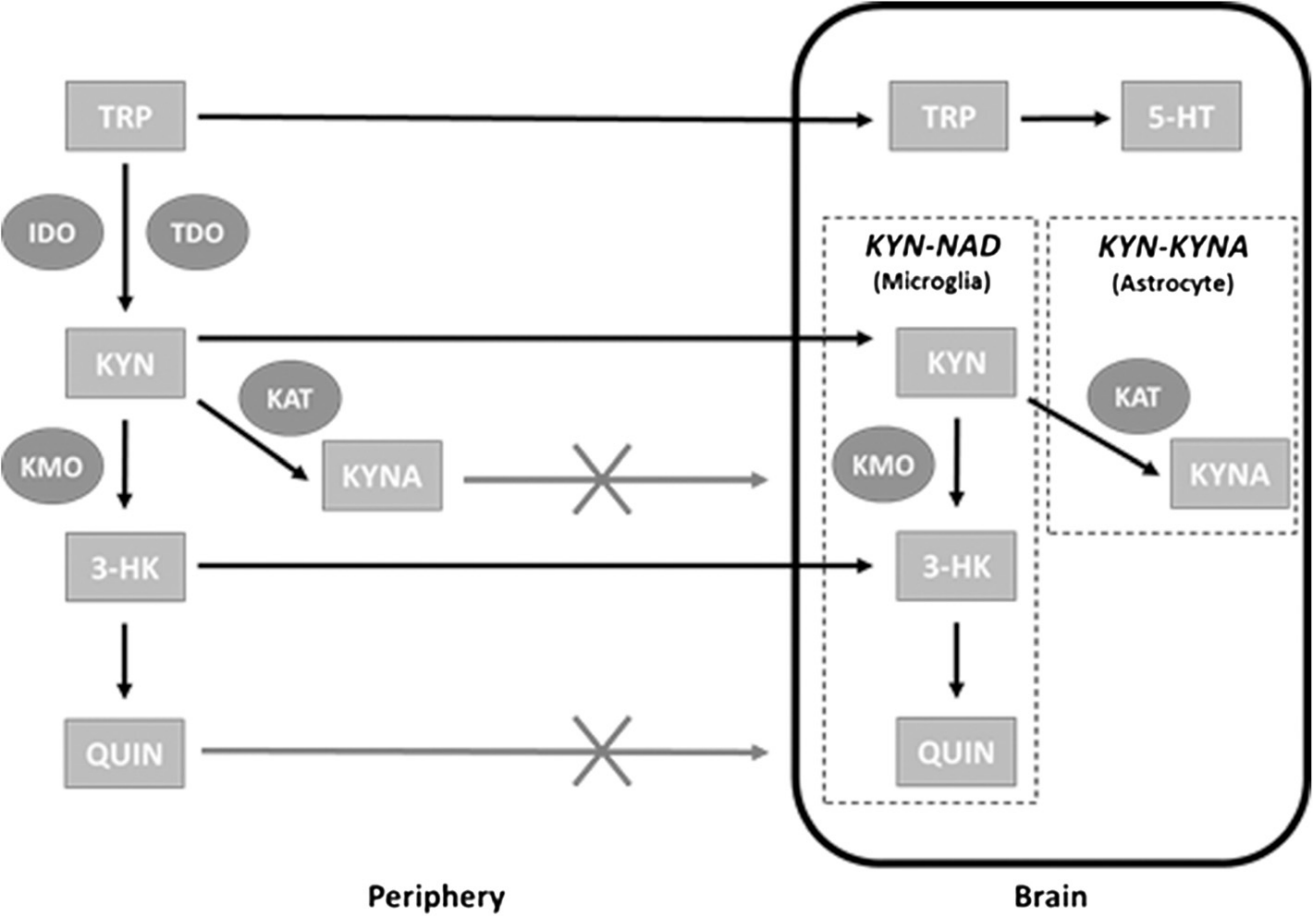
**Peripheral and central kynurenine pathway interaction.** As indoleamine 2,3-dioxygenase (IDO) and tryptophan 2,3-dioxygenase (TDO) are found at only very low concentrations within the brain, concentrations of within-brain tryptophan (TRP) and kynurenines are largely dependent on those from the periphery. TRP from the periphery competes with other large neutral amino acids (LNAA) for passage across the blood brain barrier (BBB) to be used in the synthesis of serotonin (5-HT). Kynurenine (KYN) and 3-hydroxykynurenine (3-HK) are also capable of crossing the BBB whereby they participate in the production of kynurenic acid (KYNA) and quinolinic acid (QUIN) which do not easily cross it.

The kynurenine pathway has both a peripheral and central component, and as they are not completely autonomous, the central component is heavily influenced by that of the peripheral. The rate-limiting enzyme TDO has been shown to be highly expressed within various regions of the brain including the hippocampus and cerebellum [20]. Both IDO and TDO have, however, been shown to be expressed at substantially lower levels within the brain than in the periphery making the concentration of kynurenines within the brain largely influenced by those transported across the BBB from the periphery [21]. Tryptophan, as well as the peripheral kynurenines, KYN and 3-HK are readily transported across the BBB via specialized transporters. Once in the brain, these TRP metabolites may become further degraded to produce the kynurenines KYNA and QUIN, which do not easily cross the BBB from the periphery (making within brain levels largely dependent on the synthesis from these kynurenine precursors) [22]. KYNA and QUIN are synthesized along distinct pathways within the brain due to their reliance on their respective KAT and KMO enzymes. Astrocytes possess KAT while lacking KMO thereby allowing them to participate only in the conversion of KYN to KYNA. Alternatively, microglia possess the enzyme KMO, allowing them to convert KYN to 3-HK which is later converted to QUIN via a spontaneous reaction further downstream (see Figure 2) [23]. An appropriate balance between these two branches of the kynurenine pathway is therefore critical in the maintenance of appropriate QUIN and KYNA levels within the brain.

Under healthy conditions, the peripheral component of the kynurenine pathway is well regulated, resulting in a healthy balance between peripheral concentrations of TRP and its kynurenine metabolites. As such, appropriate levels of TRP and its BBB transportable metabolites, KYN and 3-HK, are available for transportation into the brain. This allows for the synthesis of adequate levels of the neurotransmitter 5-HT, and the neuroactive kynurenines KYNA, 3-HK, and QUIN, within the brain.

### The kynurenine pathway and chronic inflammation

During a typical immune challenge, the body responds with an acute elevation in peripheral proinflammatory cytokines. Certain cytokines, such as IFN-γ, up-regulate IDO activity thereby causing an elevation in TRP degradation and an increased production of kynurenines [24,25]. As various parasites, viruses, and bacteria rely on TRP to grow and spread throughout the body, this acute response is a crucial adaptive mechanism meant to reduce TRP availability and thereby limit the spread of the pathogen [24]. During a healthy immune response, the elevation of proinflammatory cytokines is followed by an elevation in anti-inflammatory cytokines, acting to restore a balance in inflammatory mediators.

**Figure 3:**
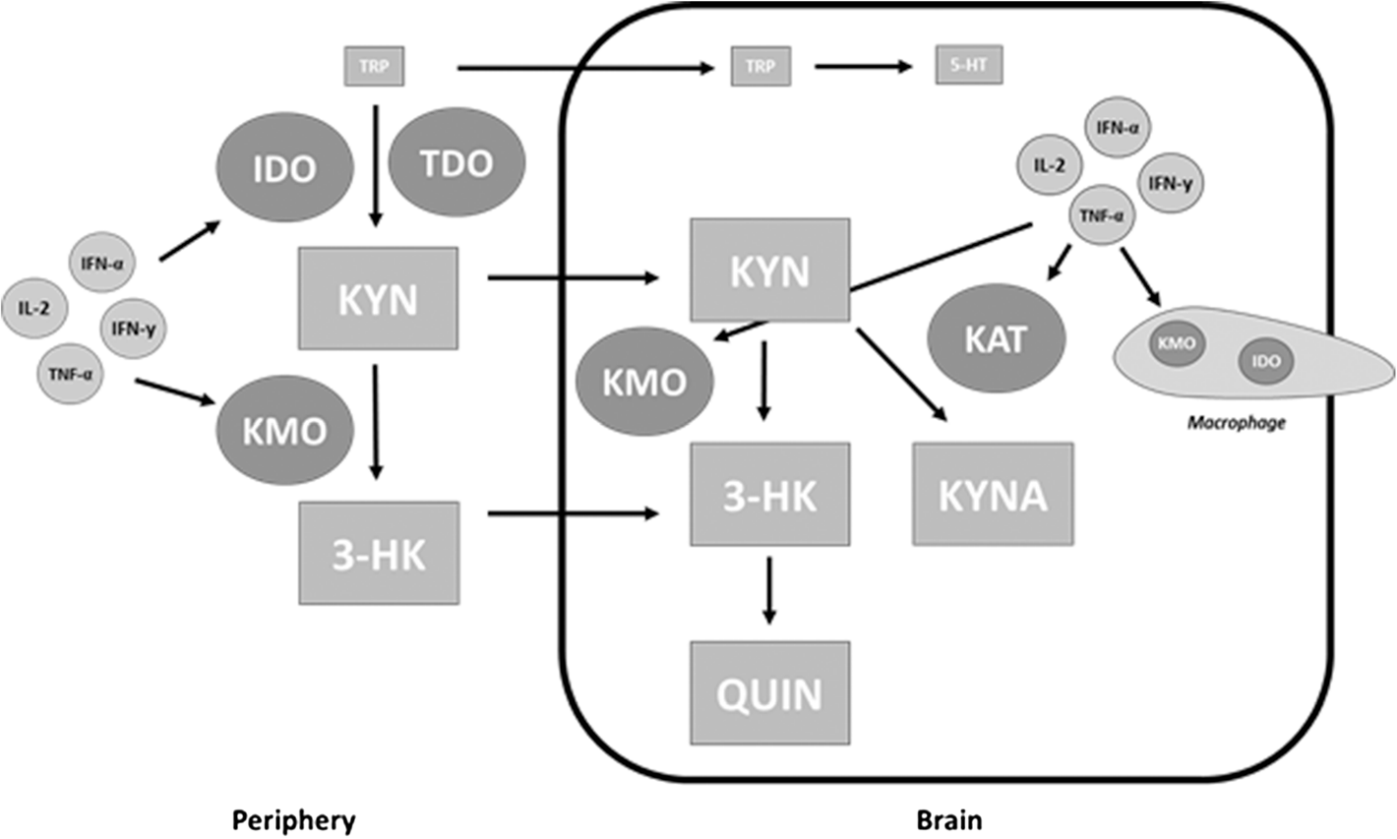
**Influence of chronic inflammation on the kynurenine pathway.** Certain proinflammatory cytokines possess the ability to up-regulate enzymes of the kynurenine pathway, thereby increasing the rate of tryptophan (TRP) degradation and production of kynurenines in the periphery. This may result in reduced concentrations of peripheral TRP and elevated peripheral levels of kynurenines. The reduced levels of peripheral TRP may then result in insufficient levels within the brain and a corresponding serotonin (5-HT) deficit. Proinflammatory cytokines also up-regulate enzymes in the brain, housed within astrocytes, microglia, and infiltrating macrophages, which utilize the elevated kynurenine concentrations for the production of further metabolites such as quinolinic acid (QUIN) and kynurenic acid (KYNA).

However, under a state of chronic inflammation, proinflammatory cytokines are maintained at a perpetually elevated state. This results in the chronic up-regulation of IDO leading to a potential lasting shift in the kynurenine pathway. This shift may lead to dramatic reductions in peripheral TRP availability as well as a surplus of peripheral kynurenines [2]. Elevated concentrations of kynurenines such as KYN, 3-HK, and QUIN have been shown to suppress T-cell proliferation and inhibit the production of TH1 cytokines [26,27]. Such an effect may partly explain the impact that TRP metabolism has on immunity under pathological conditions. A reduction in peripheral TRP concentrations may also result in a TRP deficit within the brain, thereby leading to reduced levels of 5-HT synthesis. In addition, TRP metabolites within the brain may become elevated via several mechanisms. First, the surplus of peripheral kynurenines may result in elevated levels of KYN and 3-HK within the brain due to their ability to cross the BBB. Second, during an inflammatory response, macrophages, which house key enzymes, have the ability to infiltrate the brain and thereby participate in the production of kynurenines. Lastly, as cytokines are able to cross the BBB they are able to up-regulate corresponding enzymes within astrocytes, microglia and invading macrophages resulting in an even further up-regulated production of kynurenines [28]. Together, these inflammatory-based mechanisms may result in a 5-HT deficit and surplus of kynurenines within the brain, which have important implications in symptoms of both depression and cognitive impairment (see Figure 3).

### Depression

**Figure 4:**
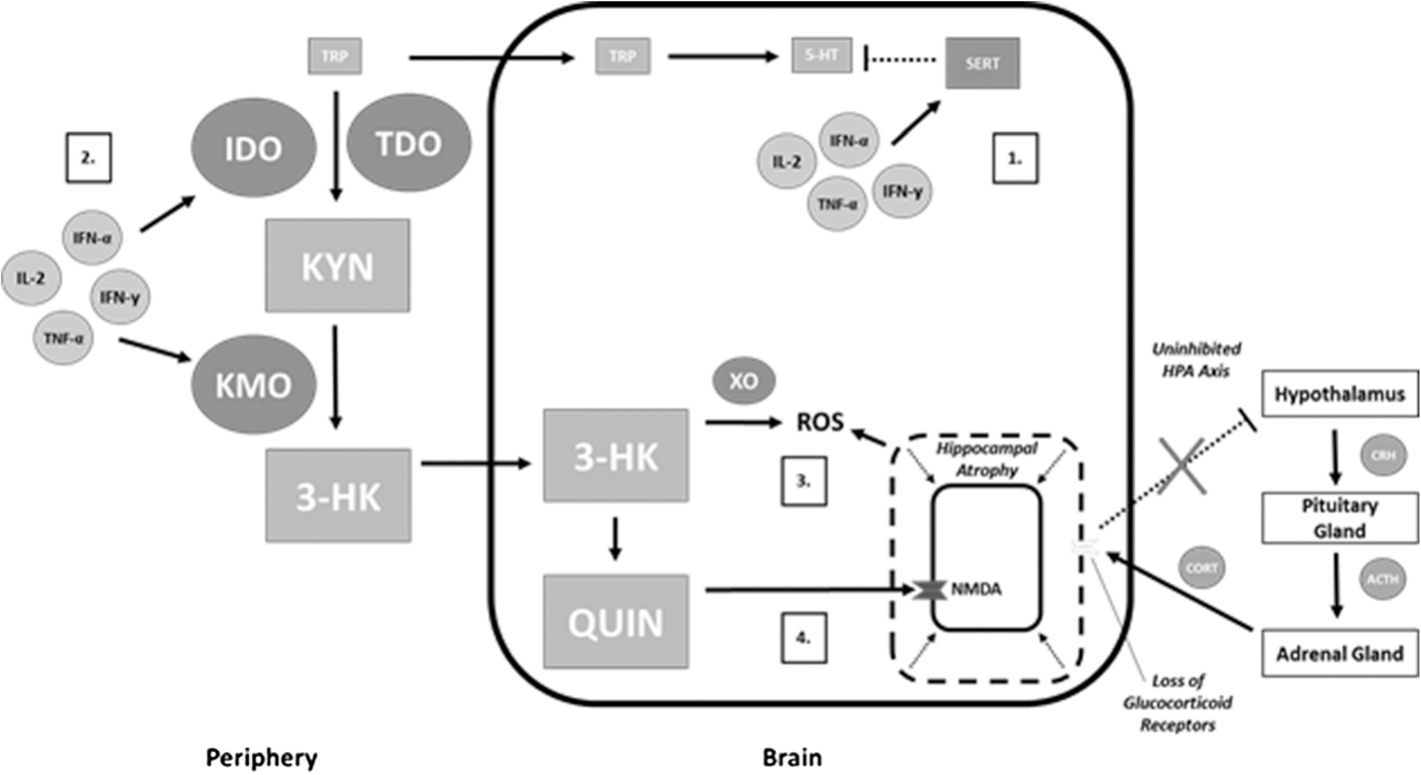
**Inflammatory mechanisms of depression.** Proinflammatory cytokines may contribute to depressive symptoms by means of various mechanisms. (1) Proinflammatory cytokines act on serotonin transporter (SERT) proteins within the brain causing a re-uptake of serotonin (5-HT) and corresponding reduced extracellular concentrations. (2) Proinflammatory cytokines up-regulate enzymes such as indoleamine 2,3-dioxygenase (IDO) and tryptophan 2,3-dioxygenase (TDO) resulting in reduced tryptophan (TRP) availability, ultimately contributing to reduced 5-HT synthesis. (3) Both 3-hydroxykynurenine (3-HK) and quinolinic acid (QUIN) may contribute to elevated levels of reactive oxygen species (ROS) and oxidative stress within the brain. (4) QUIN may induce N-methyl-D-aspartate (NMDA) over-activity thereby contributing to hippocampal atrophy and a loss of glucocorticoid receptors, ultimately leading to a loss of negative feedback and hypothalamic-pituitary-adrenal (HPA) axis over-activity.

An elevated inflammatory status may influence depressive symptoms via several potential mechanisms related to neuroactive compounds of the kynurenine pathway. First, proinflammatory cytokines such as IFN-γ, IFN-α, IL-1β and TNF-α have each been shown to directly up-regulate serotonin transporter (SERT) proteins within the brain, leading an increased reuptake of 5-HT and a corresponding reduction in extracellular concentrations [29,30]. Second, proinflammatory mediators may influence depressive symptoms by means of several indirect mechanisms associated with alterations in the activity of enzymes of the kynurenine pathway (see Figure 4).

The up-regulation of IDO and TDO and the resulting increased rate of TRP degradation may result in a TRP deficit within the periphery. This may lead to an insufficient level of TRP transportation across the BBB and ultimately contribute to a 5-HT deficit within the brain. The importance of maintaining appropriate peripheral levels of TRP have been evidenced by a number of studies in which reductions of 5-HT synthesis and relapses in depressive symptoms have been demonstrated following the transient reduction of TRP levels by means of dietary restriction [31-35]. In order to be utilized in the synthesis of 5-HT, peripheral tryptophan must compete with other large neutral amino acids (LNAA) to cross the blood brain barrier via a common transport mechanism. As an over-activation of IDO and TDO induces a selective decline in peripheral tryptophan levels, a reduction in the peripheral TRP/LNAA ratio results, thereby reducing TRP availability for the synthesis of 5-HT [19]. Therefore, under conditions of chronically elevated levels of proinflammatory cytokines the resulting alterations in enzyme activity and peripheral tryptophan levels may contribute to a 5-HT deficit within the brain (see Figure 4).

The up-regulation of IDO and TDO also results in an elevated peripheral concentration of 3-HK and QUIN. Peripheral 3-HK is able to cross the BBB and induce oxidative damage via the production of reactive oxygen species (ROS) following an interaction with the enzyme xanthine oxidase [36]. Additionally, 3-HK may be further metabolized within microglia along the KYN-NAD branch of the kynurenine pathway to produce QUIN within the brain. QUIN is a potent agonist of a glutamatergic receptor known as the N-methyl-D-aspartate (NMDA) receptor. These receptors are heavily concentrated on the hippocampus and play an important role in synaptic plasticity. When at elevated concentrations, QUIN is capable of inducing excitotoxicity by causing an over-activation of NMDA receptors leading to an increased influx of calcium ions and corresponding neuronal damage [37]. This can also lead to the production of additional free radicals and further contribute to the oxidative stress brought on by 3-HK (see Figure 4). In this way, both 3-HK and QUIN may contribute to neurodegeneration associated with depression.

The ability of elevated QUIN concentrations to cause an over-activation of NMDA receptors may also contribute to the hippocampal atrophy and HPA axis over-activity commonly reported in individuals with major depression [38-42]. The hippocampus plays an integral role in the attenuation of the HPA axis via a glucocorticoid-induced negative feedback loop. When glucocorticoids, such as cortisol, are released by the adrenal glands they act on corresponding receptors of the hippocampus, resulting in an inhibitory cascade within the HPA axis. Specifically, the activation of glucocorticoid receptors in the hippocampus results in a blunted release of corticotropin releasing hormone (CRH) from the hypothalamus, which inhibits the release of adrenocorticotropic hormone (ACTH) from the pituitary, ultimately reducing glucocorticoid release from the adrenal gland. However, overstimulation of NMDA receptors may result in hippocampal atrophy and a corresponding loss of glucocorticoid receptors. This may result in the loss of negative feedback and HPA axis over-activity (see Figure 4). An elevation in QUIN levels within the brain, as well as elevations in systemic cortisol levels, may therefore contribute to these aspects of depression. Elevated levels of both QUIN and cortisol have been demonstrated in individuals with depression [43,44].

In addition to these proposed mechanisms, several pre-clinical and clinical lines of evidence support the relationship between proinflammatory cytokines and depression. Individuals diagnosed with major depression have been consistently reported to demonstrate elevated levels of proinflammatory cytokines [13-15]. Evidence that such elevations contribute to depression via an IDO-related mechanism has also been demonstrated following cytokine therapy, utilized during human cancer trials. The acute administration of the pro-inflammatory cytokines, IL-2 and IFN-α, was shown to induce an increase in IDO activity and a corresponding reduction in both the peripheral TRP/LNAA ratio and absolute TRP concentrations [45]. Such changes in enzyme regulation and protein balances have been shown to result in the development of major depressive disorders in 15 to 40% of patients undergoing cytokine therapy with IFN-α [46]. As such, a state of chronic inflammation associated with an elevation in inflammatory mediators may contribute to symptoms of depression by means of various mechanisms associated with imbalances in the kynurenine pathway.

### Cognitive impairment

**Figure 5:**
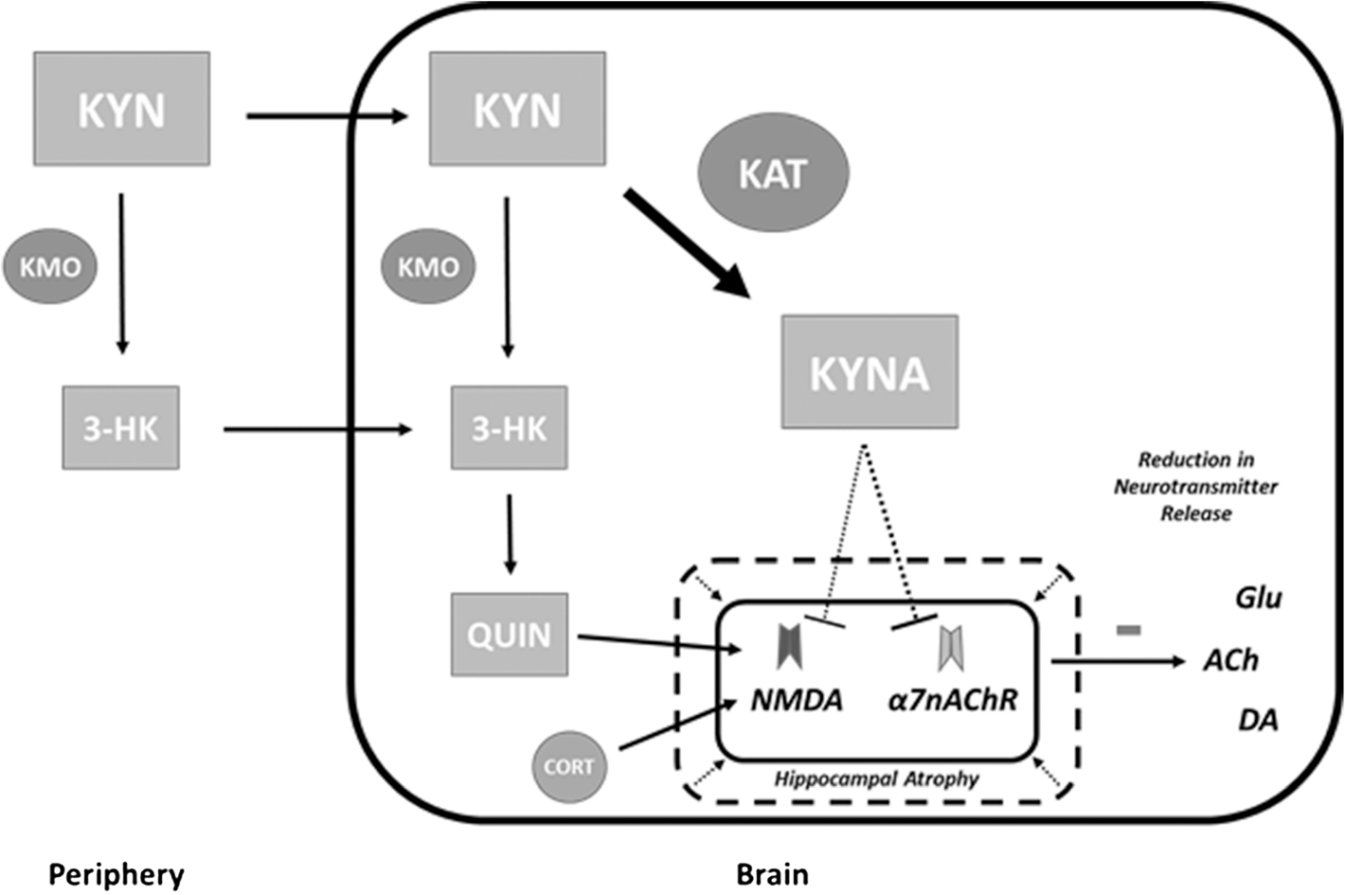
**Inflammatory mechanism of cognitive impairment.** Hippocampal atrophy caused by the over-activation of respective receptors for glucocorticoids and quinolinic acid (QUIN) may contribute to cognitive impairment. Additionally, elevated concentrations of kynurenine (KYN) that is preferentially metabolized along the kynurenine-kynurenic acid (KYN-KYNA) branch of the kynurenine pathway results in elevations of kynurenic acid (KYNA). As KYNA acts as an antagonist for both the alpha-7-nicotinic acetylcholine (α7nACh) and N-methyl-D-aspartate (NMDA) receptors it may contribute to cognitive deficits by reducing neurotransmitters such as glutamate, acetylcholine, and dopamine.

Cognitive impairment has been commonly demonstrated in depressed individuals and the severity of deficits has been shown to correlate with the number of depressive episodes experienced [47]. The relationship between depression and cognitive impairment is not fully understood; however, they may share a closely linked inflammatory etiology. The influence of chronic inflammation on hippocampal volume and HPA axis dysregulation may play an important role in the severity of each of these disorders. Hippocampal volume loss in the form of reduced grey matter density has been demonstrated in depressed individuals and has been shown to correlate with reduced scores in verbal recognition memory [48]. As previously discussed, there is evidence to support a role for chronic inflammation in the reduction in hippocampal volume involving heightened levels of metabolites and steroid hormones such QUIN and glucocorticoids. The potential apoptosis and/or inhibition of neurogenesis caused by over-activation of the hippocampus would be expected to result in cognitive deficits, due to the integral role the hippocampus plays in learning and memory. It may also lead to a vicious cycle whereby the loss of hippocampal-mediated inhibition of the HPA axis results in excess glucocorticoid production, ultimately further contributing to the extent of hippocampal atrophy (see Figure 5).

In addition to the potential structural damage, the shift in the kynurenine pathway brought on by chronic inflammation, may also contribute to cognitive deficits via reductions in neurotransmitter release. Elevated concentrations of KYN within the brain result in its metabolism along one of the two distinct branches of the kynurenine pathway. The KYN-NAD branch results in the production of 3-HK and QUIN, which has implications in the production of ROS and excitotoxicity (as previously discussed). However, the primary enzyme of the KYN-NAD branch, KMO, has been shown to be far less active in the brain in comparison to the periphery and, as such, becomes rapidly saturated by elevated levels of KYN [49]. This may result in a shift in the kynurenine pathway towards the KYN-KYNA branch and an increased production of KYNA by the more active KAT enzyme (see Figure 5).

KYNA acts as an antagonist of the alpha-7-nicotinic acetylcholine receptor (α7nAChR) and to a lesser extent, the glycine co-agonist site of the NMDA receptor [50]. Each of these receptors can be found in the hippocampus and are known to play important roles in synaptic plasticity, associated with learning and memory [51]. The inhibition of α7nACh receptors by KYNA has been shown to result in the reduced release of neurotransmitters such as glutamate, acetylcholine, and dopamine; each of which plays critical roles in cognitive processes (see Figure 5) [52-54]. Additionally, the reduction of KYNA has been demonstrated to enhance extracellular glutamate and corresponding cognitive behavior [51].

This relationship has been demonstrated using animal models whereby elevations of KYNA within the brain have been shown to induce cognitive deficits in contextual learning and memory. Whether induced indirectly, via intraperitoneal administration of kynurenine, or via direct intracerebroventricular KYNA infusion, corresponding elevations in KYNA levels within the brain have been shown to induce spatial working memory deficits and reduced orienting behavior in mice [55,56]. Similar results have been demonstrated in healthy humans whereby administration of the non-competitive NMDA glutamate receptor antagonist, ketamine, has been shown to result in reductions in verbal declarative memory [57]. Alternatively, animal models displaying low levels of KYNA have demonstrated superior cognitive performance. This has been shown using kynurenine aminotransferase II (KAT II) knockout mice, which lack the major enzyme for brain KYNA formation. These mice exhibited a 66% reduction in extracellular KYNA accompanied by a significantly increased performance in object exploration and recognition, passive avoidance, and spatial discrimination [51].

A shift in the kynurenine pathway, as well as corresponding elevated levels of inflammatory mediators, have also been demonstrated in humans afflicted with conditions associated with severe cognitive deficits. Individuals with Alzheimer’s disease have been shown to exhibit reduced peripheral tryptophan concentrations, along with heightened levels of the tryptophan metabolite, QUIN [58]. Additionally, severe elevations, of up to 25-fold, of the proinflammatory cytokine TNF-α have been demonstrated [59]. As TNF-α is a proinflammatory cytokine and potent IDO activator, it may suggest an inflammatory contribution to the molecular imbalances observed in this population. Further to these observations, the administration of the TNF-α antagonist, etanercept, has been shown to result in improved cognitive scores over a six-month administration period as well as acutely following a single dose [59,60]. Such improvements provide further evidence of a role for inflammatory mediators in cognitive processes via alterations in enzyme regulation and corresponding imbalances of critical neuroactive compounds.

### Treatment

#### Current treatment strategies

The majority of current treatment strategies aimed at improving symptoms of depression utilize drug treatments which target downstream enzymes, transporters, or receptors. Of these, selective serotonin reuptake inhibitors (SSRIs) have become the most commonly used due to their ability to induce a relatively strong and immediate alleviation of symptoms. Drug treatments of the SSRI class target serotonin transporters (SERT), and inhibit them from carrying out their role concerning 5-HT reuptake, thereby increasing extracellular levels. Use of SSRIs is however, associated with a number of side-effects and only provides transient relief of symptoms. They have also been shown to be ineffective in approximately 30% of patients [61], in whom a particularly elevated inflammatory state is typically reported [62,63]. Additionally, of individuals who do respond to treatment, an estimated 20 to 80% will relapse and experience a depressive episode within 1 to 5 years following initial treatment [64]. It may be possible that under extreme or reoccurring inflammatory episodes, the shift in the kynurenine pathway and resulting imbalance in neuroactive kynurenines, hippocampal damage and HPA axis dysfunction, may cause alterations too severe for SSRI treatment to remedy. This could partially explain the ineffectiveness of drug treatments such as SSRIs in individuals with a highly elevated inflammatory state and may suggest a need for the addition of anti-inflammatory interventions in the treatment of depression.

More recent attempts to treat cognitive impairment have focused on counteracting and/or limiting the antagonizing effects of KYNA. Agonists of the α7nACh and NMDA receptors such as galantamine have been utilized in an attempt to counteract the antagonizing role of KYNA. Such attempts have, however, produced inconclusive and only partially positive results [65-69]. It may be possible that elevated concentrations of KYNA lead to receptor saturation, thereby making it difficult to achieve any benefit from such receptor agonists. In an alternative approach, early studies examining the use of selective inhibitors for the primary enzyme involved in KYNA production, KAT II, have shown positive results in animal models. The administration of KAT II inhibitors resulted in reductions in extracellular KYNA along with elevations in glutamate, dopamine, and acetylcholine [54]. Further studies are required to examine the effectiveness of such treatments in humans as well as an appropriate dosage. As α7nACh and NMDA receptor activity has important implications in both depression and cognitive impairment, maintaining the delicate balance between receptor agonists and antagonists is critically important. As such, if either receptor agonists or KAT II inhibitors were to be used in the long-term treatment of cognitive impairment, the dose would need to be strictly controlled in order to avoid NMDA over-activity and the possibility of hippocampal atrophy and HPA axis dysregulation over time. A safer alternative may be to naturally induce molecular alterations over time via anti-inflammatory intervention strategies.

**Figure 6:**
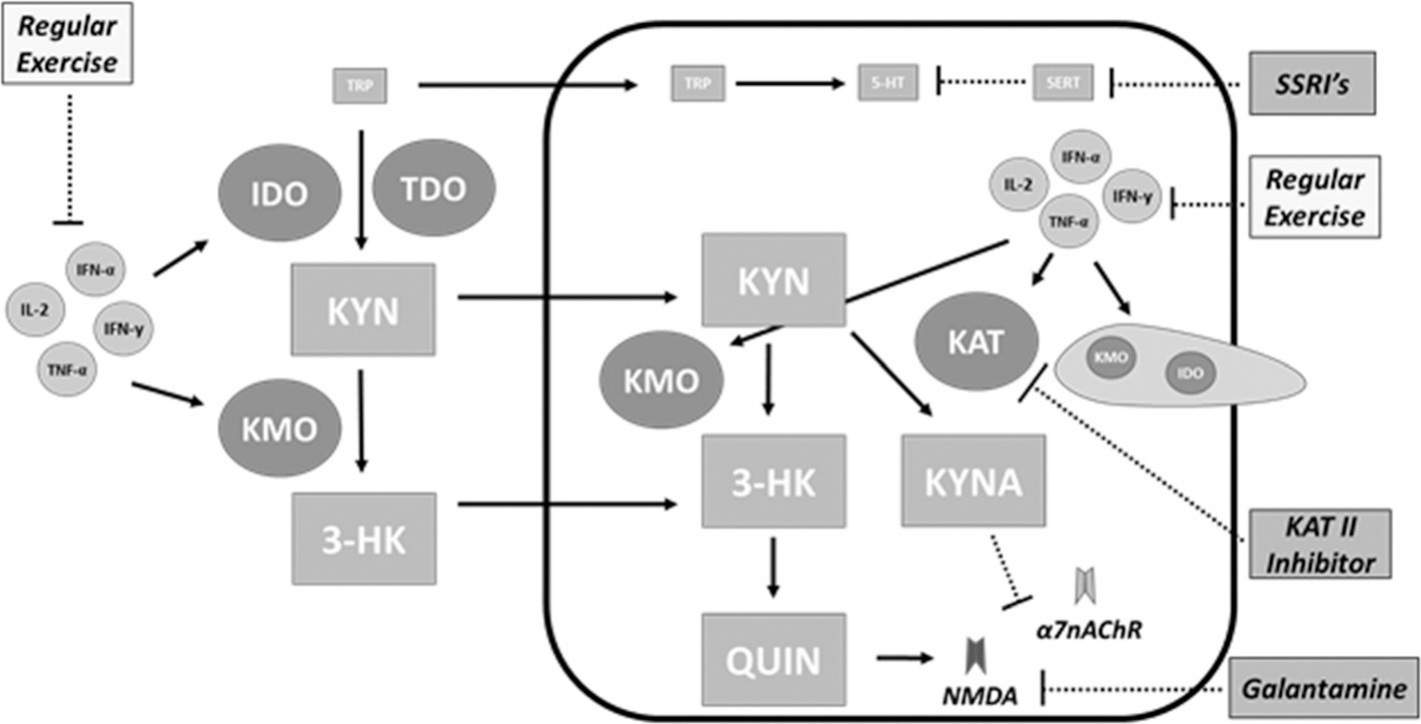
**Targets of treatment interventions.** Common treatment strategies for disorders associated with depression and cognitive impairment typically target downstream enzymes or receptors. Alternatively, intervention strategies such as regular exercise possess anti-inflammatory properties which helps restore a balance in inflammatory mediators, thereby restoring proper enzyme regulation and protein balances upstream.

Although the value of current drug treatments should not be discounted, alternative therapies which target the inflammatory basis of such disorders should also be considered. Utilizing intervention strategies which target upstream proinflammatory mediators may help to restore proper enzyme regulation and induce corresponding beneficial alterations in neuroactive compounds, thereby positively influencing a variety of disorders. Simple lifestyle alterations including the adoption of a diet consisting of foods and supplements with proven anti-inflammatory properties and participation in regular exercise may provide a safer, sustainable, and more universally applicable treatment option to that of traditional drug treatments. Although such intervention strategies do not provide the immediate effects of drug therapies, they may contribute to a more permanent solution while helping to reduce the many side-effects associated with a heavy reliance on drug interventions (Figure 6).

#### Exercise as an anti-inflammatory intervention

Regular moderate exercise has been consistently shown to reduce chronic low-grade inflammation and protect against a number of associated diseases [70,71]. Numerous cross-sectional studies have demonstrated reductions in inflammatory mediators within trained versus untrained individuals [72-75]. Although the anti-inflammatory nature of regular exercise has been well established, the mechanisms by which this is accomplished are not fully understood and are likely the result of numerous exercise-related factors.

One such theory involves a unique exercise-induced inflammatory response which differs from that of the typical response evoked by infection. Whereas infection results in the initial elevation in proinflammatory cytokines, such as TNF-α and IL-1β, it has been suggested that during an exercise-induced inflammatory response, the elevation in proinflammatory cytokines is bypassed. Instead, the initial response is a large elevation in IL-6, a cytokine with both pro and anti-inflammatory properties. This spike in IL-6 is followed by an elevation in anti-inflammatory cytokines, such as IL-10 and IL-1 receptor antagonist (IL-1RA) [70,71]. It has been suggested that IL-6 promotes an anti-inflammatory environment during exercise by promoting IL-10 and IL-1RA while inhibiting TNF-α production. This IL-6-induced inhibition of TNF-α has been demonstrated *in vitro* [76] as well as *in vivo* in animal models [77], and in humans [78]. Exercise may also inhibit proinflammatory mediators through IL-6-independent pathways via exercise-induced elevations in hormones such as epinephrine. This has been suggested due to the demonstration of TNF-α inhibition during exercise in IL-6 knockout mice [79], as well as the suppression of TNF-α following epinephrine infusion *in vivo* [80]. Whether hormonally driven, or due to a unique influence from IL-6, if acute bouts of exercise promote an anti-inflammatory environment, it may explain how exercise, when performed on a regular basis, may act to protect against a chronic low-grade inflammatory state.

Of note, many studies have demonstrated that acute bouts of exercise do in fact result in a proinflammatory response characterized by leukocytosis and elevations in proinflammatory mediators [81-83]. An acutely elevated rate of TRP metabolism and resulting increase in serum kynurenine concentrations have also been demonstrated and have been suggested to relate to a cytokine-induced up-regulation of IDO [84,85]. Further, exercise is known to result in an acute elevation in glucocorticoids [86] which have been suggested to induce the up-regulation of TDO2, further contributing to the elevated rate of TRP metabolism during exercise [87]. Despite the proposed short-term proinflammatory response, long-term metabolic and cardiovascular training adaptations are associated with a chronic reduction in inflammatory mediators which likely override the acute proinflammatory effects of exercise, resulting in a chronic shift towards an anti-inflammatory state. For example, adipose tissue is known to act as an endocrine organ, responsible for the release of a variety of proinflammatory mediators termed adipokines. As such, a reduction in adipose tissue, as would be expected over the span of a chronic training program, may result in a reduction in such proinflammatory mediators [88]. Corresponding improvements in vascular health may also protect against the plaque formation seen in atherosclerosis leading to reductions in vascular inflammation [71]. In addition, a number of other exercise-related factors including age, smoking status, hypertension, and cholesterol levels may contribute to exercise-related inflammatory benefits as each have been shown to be inversely correlated with C-reactive protein (CRP) concentrations [72].

Inconsistencies in the literature regarding the inflammatory response to an acute bout of exercise may be due to a lack of consistent exercise protocols. Differences in the mode, duration, and intensity of exercise bouts may lead to inconsistent levels of exercise-induced muscle damage, and metabolic demands resulting in variable levels of inflammatory mediators. It will be important to determine the most ideal exercise parameters to achieve the greatest inflammatory benefit if there are hopes of utilizing exercise as an effective treatment for inflammatory-based disorders. This gap in the literature may also help explain inconsistencies regarding the degree to which exercise reduces symptoms of depression and cognitive impairment. Although there is a general consensus that exercise positively impacts mood and cognition, some studies show improvements equivalent to that of traditional medications [89,90], while others show that exercise is only mildly more effective than control conditions [91]. Additionally, the mechanisms by which such benefits may be accomplished are widely debated, ranging from increased cerebral blood flow, to changes in neurotransmitter release, to actual structural changes in regions of the central nervous system (CNS), such as the hippocampus [92]. Interestingly, each of these proposed adaptations can be influenced by the inflammatory response through mechanisms previously discussed. There is a need for more methodologically robust, prospective trials with consistent exercise protocols in order to examine the true potential of exercise as a treatment strategy. Nevertheless, the anti-inflammatory properties of regular exercise have been well established, and in theory, it should help to improve or prevent symptoms related to depression and cognitive impairment by targeting upstream inflammatory processes. As such, despite the insufficient evidence to definitively state the degree to which exercise interventions may impact these disorders, it would seem there is certainly merit to implement them along with traditional drug treatments.

## Conclusion

As an inflammatory basis is becoming evident for a growing number of disorders, therapies which target the immune system and act to restore a balance in inflammatory mediators should be considered as potential methods of treatment and prevention. Although the effectiveness of current drug treatments should not be undervalued, pairing them with intervention strategies that target upstream inflammatory processes may enhance the outcome of drug treatments for disorders impacted by chronic inflammation. It is difficult to make definitive conclusions regarding the degree to which exercise, as an anti-inflammatory strategy, influences conditions such as depression and cognitive impairment, as previous inferences are largely based on observational studies with highly variable protocols. However, the inflammatory etiology behind such disorders and the anti-inflammatory mechanisms of exercise have been well established and, therefore, should in theory provide some benefit. Additionally, given the minimal side-effects associated with such lifestyle alterations, as well as the potential for a variety of other health benefits, there is seemingly little reason not to promote exercise as a treatment option. Whether exercise has the potential to be effective as a stand-alone treatment, or would need to be utilized in conjunction with common drug treatments, is currently unclear and will likely vary between patients depending upon a number of factors. Further research will also be needed to determine the most appropriate exercise parameters in order to achieve the greatest anti-inflammatory benefit. A greater emphasis on intervention strategies which target inflammation may provide stronger and more sustainable improvements by targeting the inflammatory mediators initially responsible for the alterations in enzyme regulation which ultimately contribute to a number of disorders. Such strategies may help to reduce symptoms of disorders such as depression and cognitive impairment while avoiding the many side-effects associated with a heavy reliance on current drug treatments.

## Abbreviations

3-HK: 3-hydroxykynurenine
5-HT: serotonin
α7nAChR: alpha-7-nicotinic acetylcholine receptor
ACh: acetylcholine
ACTH: adrenocorticotropic hormone
BBB: blood brain barrier
CORT: cortisol
CRH: corticotropin releasing hormone
CRP: C reactive protein
DA: dopamine
Glu: glutamate
HPA axis: hypothalamic-pituitary-adrenal axis
IDO: indoleamine 2,3-dioxygenase
IFN: interferon
IL: interleukin
IL-1RA: interleukin-1 receptor antagonist
KAT: kynurenine aminotransferase
KAT II: kynurenine aminotransferase II
KMO: kynurenine 3-monooxygenase
KYN: kynurenine
KYNA: kynurenic acid
KYN-KYNA: kynurenine-kynurenic acid
KYN-NAD: kynurenine-nicotinamide adenine dinucleotide
LNAA: large neutral amino acid
NMDA: N-methyl-D-aspartate
QUIN: quinolinic acid
ROS: reactive oxygen species
SERT: serotonin transporter
SSRIs: selective serotonin reuptake inhibitors
TDO: tryptophan 2,3-dioxygenase
TNF: tumor necrosis factor
TRP: tryptophan
